# Importance of Quality Assessment in Clinical Research in Japan

**DOI:** 10.3389/fphar.2019.01228

**Published:** 2019-10-18

**Authors:** Rieko Ueda, Yuji Nishizaki, Yasuhiro Homma, Shoji Sanada, Toshiaki Otsuka, Shinji Yasuno, Kotone Matsuyama, Naotake Yanagisawa, Masashi Nagao, Kazutoshi Fujibayashi, Shuko Nojiri, Yumiko Seo, Natsumi Yamada, Patrick Devos, Hiroyuki Daida

**Affiliations:** ^1^Medical Technology Innovation Center, Juntendo University, Tokyo, Japan; ^2^Department of Cardiovascular Medicine, Juntendo University Graduate School of Medicine, Tokyo, Japan; ^3^Department of Orthopedic Surgery, Juntendo University School of Medicine, Tokyo, Japan; ^4^Department of Medical Innovation, Osaka University Hospital, Suita, Japan; ^5^Department of Cardiovascular Medicine, Osaka University Graduate School of Medicine, Suita, Japan; ^6^Center for Clinical Research and Innovation, Osaka City University Hospital, Osaka, Japan; ^7^Department of Hygiene and Public Health, Nippon Medical School, Tokyo, Japan; ^8^Center for Clinical Research, Nippon Medical School Hospital, Tokyo, Japan; ^9^Clinical Research Support Center, The Jikei University School of Medicine, Tokyo, Japan; ^10^Center for Strategic Research Initiative, Nippon Medical School, Tokyo, Japan; ^11^Department of Health Policy and Management, Nippon Medical School, Tokyo, Japan; ^12^School of Health and Sports Science, Juntendo University, Chiba, Japan; ^13^Department of General Medicine, Juntendo University Graduate School of Medicine, Tokyo, Japan; ^14^Univ. Lille, CHU Lille, Lille, France; ^15^Faculty of Health Science, Juntendo University, Tokyo, Japan

**Keywords:** clinical trial, core clinical research hospital, journal impact factor, quality assessment, SIGAPS

## Abstract

**Background**: The number of papers published by an institution is acknowledged as an easy-to-understand research outcome. However, the quantity as well as the quality of research papers needs to be assessed.

**Methods:** To determine the relation between the number of published papers and paper quality, a survey was conducted to assess publications focusing on interventional clinical trials reported by 11 core clinical research hospitals. A score was calculated for each paper using *Système d’interrogation*, *de gestionet d’analyse des publications scientifiques* scoring system, allowing for a clinical paper quality assessment independent of the field. Paper quality was defined as the relative Journal impact factor (IF) total score/number of papers.

**Results:** We surveyed 580 clinical trial papers. For each of the 11 medical institutions (a–k), respectively, the following was found: number of published papers: a:66, b:64, c:61, d:56, e:54, f:51, g:46, h:46, i:46, j:45, k:45 (median: 51, maximum: 66, minimum: 45); total Journal IF: a:204, b:252, c:207, d:225, e:257, f:164, g:216, h:190, i:156, j:179, k:219 (median: 207, maximum: 257, minimum: 156); relative Journal IF total score: a:244, b:272, c:260, d:299, e:268, f:215, g:225, h:208, i:189, j:223, k:218 (median: 225, maximum: 299, minimum: 189); and paper quality (relative Journal IF total score/number of papers): a:3.70, b:4.25, c:4.26, d:5.34, e:4.96, f:4.22, g:4.89, h:4.52, i:4.11, j:4.96, k:4.84 (median: 4.52, maximum: 5.34, minimum: 3.70). Additionally, no significant relation was found between the number of published papers and paper quality (correlation coefficient, −0.33, P = 0.32).

**Conclusions:** The number of published papers does not correspond to paper quality. When assessing an institution’s ability to perform clinical research, an assessment of paper quality should be included along with the number of published papers.

## Background

Assessing a medical institution’s ability to perform clinical research is extremely important. The number of scientific papers published by an institution is acknowledged as an easy-to-understand research outcome. However, not only the quantity but also the quality of papers needs to be addressed. One of the most well-established metric indicators for assessing paper quality is the impact factor (IF) of the journal in which the article has been published (Garfield, 2006). However, citation frequency and trends vary across research fields, and the inability to compare the IF of journals from different scientific areas is the main shortcoming of this metric. To address this issue, the bibliometric software tool developed in France, *Système d’interrogation, de gestionet d’analyse des publications scientifiques* (SIGAPS; “software to identify, manage, and analyze scientiﬁc publications”), is used to calculate a score that objectively assesses paper quality (Devos et al., 2003; Devos et al., 2006; Devos, 2008), allowing a comparison of the IF of journals from different research fields using the SIGAPS estimated score. This score is therefore considered a journal’s “relative IF score.” To estimate the SIGAPS score, the IFs of journals from a specific research field are ranked from high to low and points are attributed based on the IF percentiles, providing a quality assessment that is independent of the research field.

For this study, 580 clinical trial papers were surveyed to investigate the relation between number of papers and paper quality (relative Journal IF total score/number of papers). Clinical trial papers were retrieved from 11 core clinical research hospitals in Japan.

## Methods

### Study Design

Based on data published on the website of the Ministry of Health, Labour and Welfare, a descriptive research was conducted to compare the quantity and quality of clinical trial papers published by 11 core clinical research hospitals. A relative Journal IF score, based on the SIGAPS scoring system, was used to assess paper quality.

### Core Clinical Research Hospitals

In Japan, core clinical research hospitals are appointed by the Medical Care Act. They play a central role in physician-led clinical trials and in clinical research developed according to international standards toward the development of innovative pharmaceuticals and medical instruments in Japan. These hospitals support clinical research developed in other medical institutions and play a key role in optimizing next-generation healthcare by enhancing the quality of clinical research in those medical institutions where joint research efforts are conducted.

The Ministry of Health, Labour and Welfare grants approval for core clinical research hospitals (Ministry of Health, Labour and Welfare, 2017), which are required to meet specific requirements established by the Medical Care Act. Approval requirements include the existence of infrastructures to support clinical research, both in terms of facilities and personnel, as well as evidence of former clinical research performance. Moreover, each of these hospitals is strictly audited based on on-site inspections. This system was established in April 2015 and, as of February 2019, 12 medical institutions (nine national universities, two national centers, and one private university) have been granted formal approval.

### Examination of Clinical Trial Papers

To meet former clinical research performance requirements, core hospitals must have submitted a minimum of 45 clinical trial papers published over the last three years. All papers were required to be published in PubMed. Requirements for approval of hospitals as core clinical research institutions, as determined by the Ministry of Health, Labour and Welfare, only include the development of interventional clinical trials, excluding observational studies.

For study purposes, all clinical trial papers submitted until November 2018 by 11 core hospitals were extracted from the Ministry of Health, Labour and Welfare website and examined. Additionally, a list of clinical trial publications from each study hospital was retrieved from a 2017 business report of core clinical research hospitals published by the Ministry of Health, Labour and Welfare (Ministry of Health, Labour and Welfare, 2017).

### Identifying Relevant Journals

Using PubMed “JournalTitle,” “MedAbbr,” and “IsoAbbr,” search strings, both printed and online International Standard Serial Number information was obtained by comparing journals in which papers listed in the business report were published. If a match between PubMed records and journal list content could not be found, a visual check was conducted whenever appropriate.

### Method to Calculate Relative Journal Impact Factor Score

Relative Journal IF score was calculated based on the SIGAPS scoring system. Research fields of each clinical trial papers were initially categorized based on the Web of Science Category. A Journal IF percentile was then calculated for each field, and both a rank and a score (relative Journal IF score) were attributed to each journal based on that percentile (**Table 1**). This Journal IF percentile was applied to the 2018 release of Clarivate Analytics’ Journal Citation Reports (Journal Citation Reports 2017 Metrics). A formula of Journal IF percentile was as follows: Journal IF percentile = (N − R + 0.5)/N, wherein N was the number of journals in the category and R was the Descending Rank.

**Table 1:** Journal rank and score (relative Journal IF score) based on SIGAPS.

Journal IF Percentile	Rank	Score (relative Journal IF score)
≥90	A	8
75– < 90	B	6
50– < 75	C	4
25– < 50	D	3
<25	E	2
Not applicable to Journal citation reports (no IF)	NC	1

IF, impact factor; SIGAPS, Système d’interrogation, de gestionet d’analyse des publications scientifiques; NC, not categorized.

For journals with multiple IF percentiles, the highest value was selected. Additionally, paper quality was defined as relative Journal IF total score/number of papers.

SIGAPS components include the journal’s rank and the author’s rank, including first or last author (4 points), second or second-to-last author (3 points), third author (2 points), or any other contributing author (1 point) with a weighting factor. However, to approve a clinical research hospital, the Ministry of Health, Labour and Welfare requires that the first author of a clinical publication belongs to the considered institution. For this reason, in this study the relative Journal IF score was calculated based on only the journal’s rank.

**Table 2** shows journal names, category descriptions, Journal IF percentiles, ranks, and scores (relative Journal IF score) for 56 papers published by clinical researchers at Hospital D.

**Table 2:** Example of relative Journal IF score calculated using the SIGAPS scoring system (Hospital D).

No.	Journal	Category Description	Journal IF Percentile	Rank	Score (relative Journal IF)
1	International Wound Journal	DERMATOLOGY	65.873	C	4
2	JOURNAL OF BONE AND MINERAL METABOLISM	MEDICINE, RESEARCH & EXPERIMENTAL	46.241	D	3
3	CURRENT MEDICAL RESEARCH AND OPINION	MEDICINE, GENERAL & INTERNAL	76.948	B	6
4	STROKE	PERIPHERAL VASCULAR DISEASE	94.615	A	8
5	INTERNAL MEDICINE	MEDICINE, GENERAL & INTERNAL	26.299	D	3
6	AMERICAN JOURNAL OF OPHTHALMOLOGY	OPHTHALMOLOGY	90.678	A	8
7	Blood Cancer Journal	ONCOLOGY	91.216	A	8
8	Tissue Engineering Part B: Reviews	–	–	NC	1
9	SUPPORTIVE CARE IN CANCER	REHABILITATION	83.846	B	6
10	International Journal of Clinical Oncology	ONCOLOGY	36.712	D	3
11	EXPERT OPINION ON PHARMACOTHERAPY	PHARMACOLOGY & PHARMACY	74.904	C	4
12	RADIOTHERAPY AND ONCOLOGY	RADIOLOGY, NUCLEAR MEDICINE & MEDICAL IMAGING	88.672	B	6
13	INVESTIGATIVE OPHTHALMOLOGY & VISUAL SCIENCE	OPHTHALMOLOGY	85.593	B	6
14	INVESTIGATIVE OPHTHALMOLOGY & VISUAL SCIENCE	OPHTHALMOLOGY	85.593	B	6
15	CLINICAL DRUG INVESTIGATION	PHARMACOLOGY & PHARMACY	30.843	D	3
16	BMC Medicine	MEDICINE, GENERAL & INTERNAL	93.831	A	8
17	Journal of Translational Medicine	MEDICINE, RESEARCH & EXPERIMENTAL	79.323	B	6
18	CANCER CHEMOTHERAPY AND PHARMACOLOGY	PHARMACOLOGY & PHARMACY	59.195	C	4
19	PLoS One	MULTIDISCIPLINARY SCIENCES	77.344	B	6
20	CANCER CHEMOTHERAPY AND PHARMACOLOGY	PHARMACOLOGY & PHARMACY	59.195	C	4
21	Journal of Diabetes Investigation	ENDOCRINOLOGY & METABOLISM	54.196	C	4
22	MEDICAL PHYSICS	RADIOLOGY, NUCLEAR MEDICINE & MEDICAL IMAGING	72.266	C	4
23	INTERNATIONAL JOURNAL OF COLORECTAL DISEASE	SURGERY	69.75	C	4
24	JOURNAL OF THE AMERICAN GERIATRICS SOCIETY	GERONTOLOGY	95.833	A	8
25	Journal of Hepato-Biliary-Pancreatic Sciences	SURGERY	77.75	B	6
26	EXPERT OPINION ON PHARMACOTHERAPY	PHARMACOLOGY & PHARMACY	74.904	C	4
27	BMJ Open	MEDICINE, GENERAL & INTERNAL	72.403	C	4
28	INVESTIGATIVE OPHTHALMOLOGY & VISUAL SCIENCE	OPHTHALMOLOGY	85.593	B	6
29	PLoS One	MULTIDISCIPLINARY SCIENCES	77.344	B	6
30	Journal of Diabetes Science and Technology	–	–	NC	1
31	JOURNAL OF CLINICAL ONCOLOGY	ONCOLOGY	98.423	A	8
32	JOURNAL OF SURGICAL ONCOLOGY	SURGERY	78.25	B	6
33	Clinical Genitourinary Cancer	UROLOGY & NEPHROLOGY	62.5	C	4
34	JOURNAL OF GLAUCOMA	OPHTHALMOLOGY	38.136	D	3
35	HEART AND VESSELS	CARDIAC & CARDIOVASCULAR SYSTEMS	46.484	D	3
36	EUROPEAN SPINE JOURNAL	ORTHOPEDICS	74.675	C	4
37	Scientific Reports	MULTIDISCIPLINARY SCIENCES	82.031	B	6
38	Cardiovascular Diabetology	ENDOCRINOLOGY & METABOLISM	84.965	B	6
39	Journal of Atherosclerosis and Thrombosis	PERIPHERAL VASCULAR DISEASE	63.846	C	4
40	ANNALS OF SURGICAL ONCOLOGY	SURGERY	90.25	A	8
41	INTERNATIONAL ORTHOPAEDICS	ORTHOPEDICS	66.883	C	4
42	AMERICAN JOURNAL OF OPHTHALMOLOGY	OPHTHALMOLOGY	90.678	A	8
43	INVESTIGATIVE OPHTHALMOLOGY & VISUAL SCIENCE	OPHTHALMOLOGY	85.593	B	6
44	PEDIATRIC BLOOD & CANCER	PEDIATRICS	79.435	B	6
45	GASTROINTESTINAL ENDOSCOPY	GASTROENTEROLOGY & HEPATOLOGY	89.375	B	6
46	AMERICAN JOURNAL OF OPHTHALMOLOGY	OPHTHALMOLOGY	90.678	A	8
47	AURIS NASUS LARYNX	OTORHINOLARYNGOLOGY	37.805	D	3
48	INTERNATIONAL JOURNAL OF COLORECTAL DISEASE	SURGERY	69.75	C	4
49	AMERICAN JOURNAL OF OPHTHALMOLOGY	OPHTHALMOLOGY	90.678	A	8
50	JOURNAL OF BIOMEDICAL OPTICS	OPTICS	64.362	C	4
51	Journal of Applied Physiology (1985)	SPORT SCIENCES	87.037	B	6
52	JACC-Cardiovascular Interventions	CARDIAC & CARDIOVASCULAR SYSTEMS	93.359	A	8
53	LEUKEMIA	HEMATOLOGY	95.07	A	8
54	GASTROINTESTINAL ENDOSCOPY	GASTROENTEROLOGY & HEPATOLOGY	89.375	B	6
55	Scientific Reports	MULTIDISCIPLINARY SCIENCES	82.031	B	6
56	RETINA-THE JOURNAL OF RETINAL AND VITREOUS DISEASES	OPHTHALMOLOGY	88.983	B	6

IF, impact factor; SIGAPS, Système d’interrogation, de gestionet d’analyse des publications scientifiques.

### Statistical Analysis

The correlation between paper quantity and quality was estimated through the Spearman’s correlation coefficient. Aggregation and analysis of all data was performed using SAS ver. 9.4 (SAS Institute Inc., Cary, NC, USA).

## Results

Overall, 580 clinical trial publications from the last three years were surveyed. The number of published papers, total Journal IF, relative Journal IF total score, and paper quality (relative Journal IF total score/number of papers) for each of the 11 medical institutions investigated are shown in **Table 3**.

**Table 3:** Quality of papers by medical institution.

Hospital	a	b	c	d	e	f	g	h	i	j	k	Median	Maximum	Minimum
Number of papers	66	64	61	56	54	51	46	46	46	45	45	51	66	45
Total Journal IF	204	252	207	225	257	164	216	190	156	179	219	207	257	156
Relative Journal IF total score	244	272	260	299	268	215	225	208	189	223	218	225	189	299
Paper quality	3.70	4.25	4.26	5.34	4.96	4.22	4.89	4.52	4.11	4.96	4.84	4.52	3.70	5.34

IF, impact factor; Paper quality = relative Journal IF total score/number of papers.

**Figure 1:**
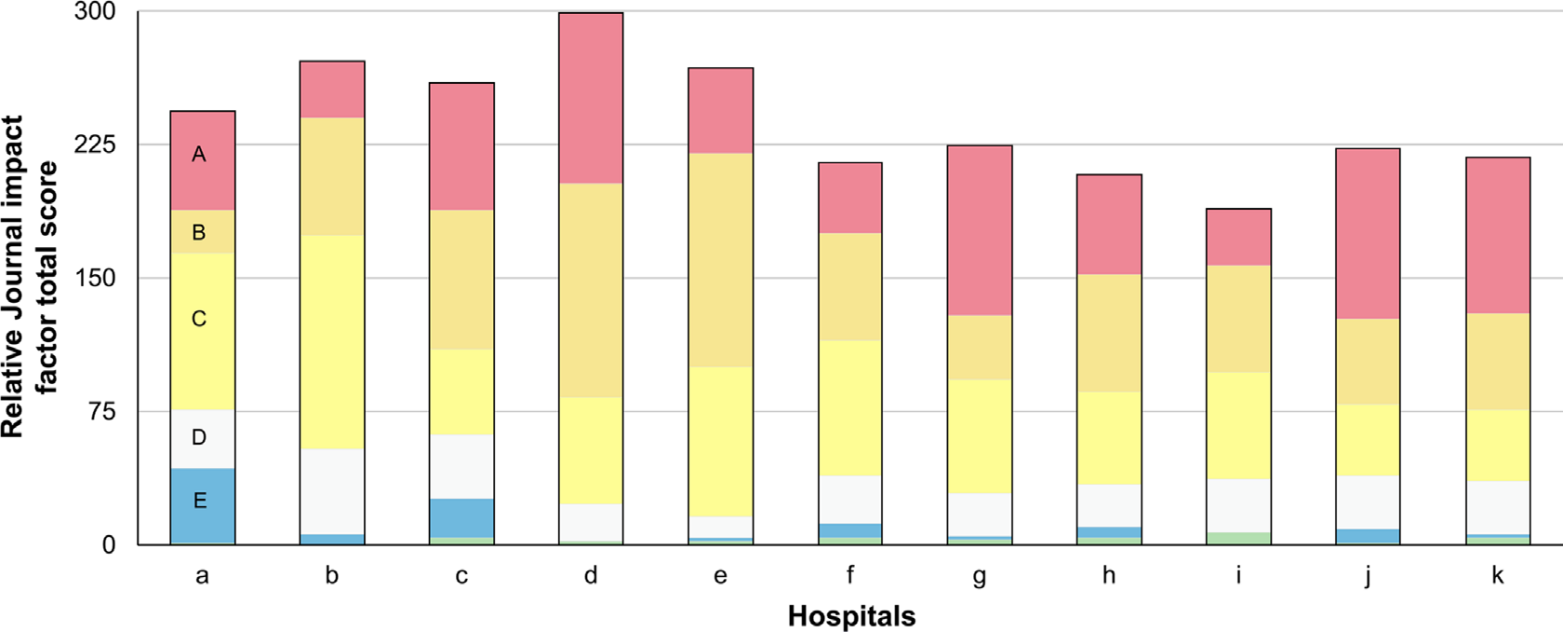
Distribution of journal rank by medical institution.

**Figure 1** depicts journal rank distribution based on Journal IF percentile for each medical institution.

**Figure 2:**
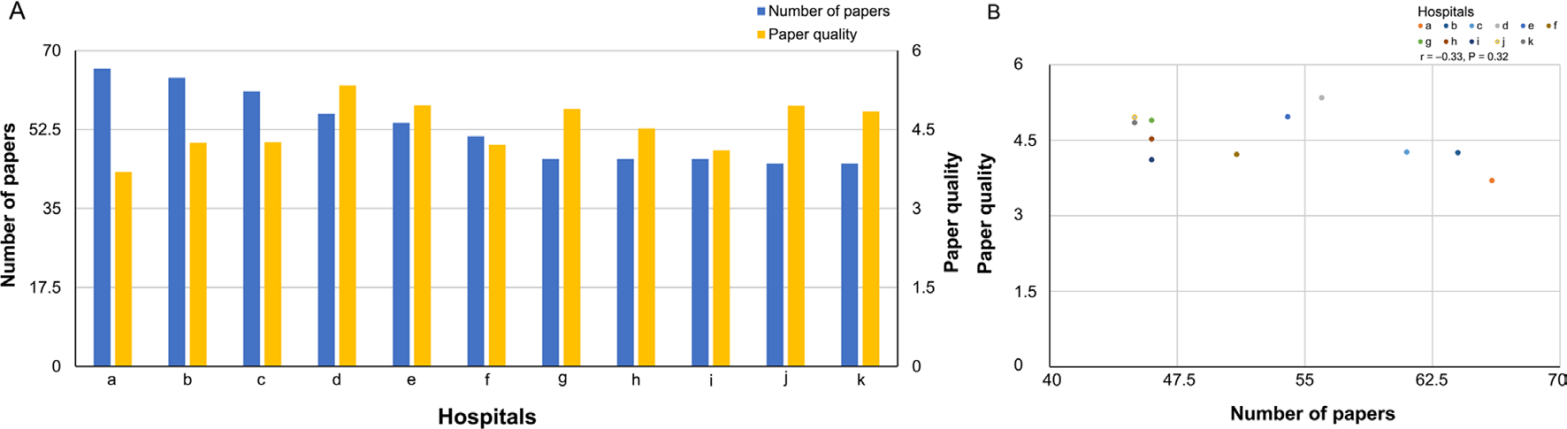
**(A)** A comparison of number of published papers and paper quality (bar graph). **(B)** A comparison of number of published papers and paper quality (scatter plot).

A comparison between the number of published papers and paper quality is shown in **Figure 2A** (bar graph) and **Figure 2B** (scatter plot). Spearman’s correlation showed that the number of published papers did not correlate with paper quality (correlation coefficient, −0.33, P = 0.32). Additionally, no significant association was found between the number of published papers and paper quality as calculated by the absolute Journal IF (total Journal IF/number of papers) (correlation coefficient, −0.53, P = 0.09).

We performed additional analyses with data excluding 44 protocol papers. The following was found: number of published papers: a:41, b:56, c:61, d:54, e:54, f:51, g:45, h:45, i:40, j:44, k:45 (median: 45, maximum: 61, minimum:40); total Journal IF: a:166, b:221, c:207, d:218, e:257, f:164, g:213, h:188, i:147, j:175, k:219 (median: 207, maximum: 257, minimum:147); relative Journal IF total score: a:180, b:243, c:260, d:289, e:268, f:215, g:221, h:205, i:172, j:219, k:218 (median: 219, maximum: 289, minimum: 172); and paper quality (relative Journal IF total score/number of papers): a:4.39, b:4.34, c:4.26, d:5.35, e:4.96, f:4.22, g:4.91, h:4.56, i:4.30, j:4.98, k:4.84 (median: 4.56, maximum: 5.35, minimum: 4.22). No significant relation was also found between the number of published papers and paper quality (correlation coefficient, −0.08, P = 0.80).

## Discussion

In this study, 580 clinical trial papers reported by 11 core clinical research hospitals in Japan were surveyed to examine the relation between quantity and quality of publications. Results showed no significant relation between the number of papers published by a hospital and the quality of those papers. Therefore, an evaluation of both the number and quality of published papers should be performed when assessing an institution’s competence to execute clinical research based on their scientific publications.

This study employed a quality assessment metric indicator (relative Journal IF score) calculated based on the SIGAPS scoring system. SIGAPS was developed in 2002 at the French University Hospital, Lille (CHU) (Devos et al., 2003; Devos et al., 2006; Devos, 2008). The SIGAPS score is one of the metric indicators used by the French Ministry of Health when allocating research funds to public research institutions such as university hospitals (Rouvillain et al., 2014) In France, several studies evaluated surgery and internal medicine scientific publication outputs using the SIGAPS scoring system (Rouprêt et al., 2012; Lefèvre et al., 2013). Griffon et al. evaluated that association between the SIGAP score and publications in French (Griffon et al., 2012). Additionally, several researchers have discussed how to use the SIGAP score in several fields (Sabourin and Darmoni, 2008; Mancini et al., 2009; Darmoni et al., 2009; Ruffion et al., 2012). The present study represents the first effort toward the development of a quality assessment method for core clinical research hospitals in Japan using a SIGAPS-based score: the relative Journal IF score.

In this study, the only paper quality assessment measure used was the relative Journal IF score. However, other metrics must be considered. The citation index is one such candidate (Garfield, 2006). However, the year of publication and the research field impact associated with this metric should be considered, and a method that corrects for these factors is required. As an example, the authors propose to use the percentage of publications in top 1% or top 10%, referring to the percentage of published papers within the same research field and year falling in the top 1% or top 10% of papers with the highest number of citations. Moreover, h-index is a useful metric indicator for measuring both paper productivity and impact at the author level (Hirsch, 2007; Allen et al., 2009; Durieux and Gevenois, 2010). PubMed currently provides citation indexes and impact metrics, such as the Relative Citation Ratio (Hutchins et al., 2016). The next phase of this research comprises a multidimensional clinical paper assessment through the use of a composite metric combining the relative IF, citation index, and h-index. Moreover, unlike IF and citation index, an advantage of altmetrics is that it can be evaluated instantaneously, eliminating the need to wait till the article is published. In addition, almetrics can provide a comprehensive overview of the degree of impact on society by incorporating aspects such as the social impact of publications, including media and mass media references. Based on both the aforementioned points, introducing altmetrics to the evaluation index could prove to be of considerable value.

When assessing the quality of medical publications, it is also necessary to account for their contribution to the development of treatment guidelines. Medical treatment guidelines are developed by field specialists, and they provide the latest evidence-based data on medical practices and procedures in the clinical setting. Areas covered ranged from disease pathophysiology to prevention, diagnosis, treatment, and rehabilitation, and guidelines in each of these areas contribute to improve quality standards of medical treatment. Medical treatment guidelines are important contributions to healthcare, and publications focusing them should be considered as high-quality, regardless of the journal’s IF or citation index.

The method for calculating relative Journal IF based on the SIGAPS scoring system that was developed in France is clear and technically acceptable in any country other than France, as described in the Methods section. However, there are issues regarding Journal IF that still need to be solved. One challenge is the adequate assessment of negative studies. For example, clinical researchers tend not to publish negative studies with small sample sizes. This could cause publication bias. Since negative studies with small sample sizes may have a very high social significance, there is a need to create a mechanism to appropriately incorporate the value of negative studies with small sample sizes in Journal IF.

Although the focus of this study was paper quality assessment, it is equally important to develop objective metric indicators for quality assessment of clinical research itself. Months or years can go between completion of clinical studies and publication of their results. Therefore, in absence of a metric indicator for paper quality assessment, quality assessment of the ongoing or recently completed clinical study is not possible. Developing metric indicators to assess the quality of clinical research, and not only the quality of resulting publications, is therefore an unmet and urgent need. As the next step, an index should be added that can objectively evaluate the quality of the research process, including the number of patients enrolled, speed of enrollment, rate of satisfaction with enrollment, number of protocol deviations, and number of protocol amendments.

The present study has several limitations, including undeniable social issues. The first limitation is the method used to calculate the relative Journal IF score. This method attributes a score by categorizing a paper based on the percentiles for its field, and a concern exists that the maximum and minimum Journal IF in the same percentile may be treated as the same score.

The second limitation relates to policy differences for assessment of track records of research institutions, which constitutes an undeniable social issue. When assessing faculty track records, some research institutions may prioritize paper quality over the number of published papers, others may take the opposite approach, and others may even prioritize citations instead. Overcoming these inconsistencies will require the development of metric indicators combining number of papers and relative Journal IF scores with citation indexes or h-indexes in a well-balanced fashion.

The third limitation is the impact of research fields considered, at certain times, as “fashionable.” This also constitutes an indisputable social issue. At different time periods, research in popular fields may register great advances, and government agencies and foundations tend to apply their budgets accordingly. Research institutions that can adapt to such trends are likely to experience an increase, both in the number of published papers, as in paper quality. Counteracting this trend-driven impact is difficult. For example, in the field of rare and incurable diseases, the Government of Japan’s “Act on Medical Care for Patients with Intractable Diseases,” passed on May 23, 2014, promoted a reform and the establishment of a sustainable social insurance system. The law went into effect on January 1, 2015, using consumption taxes to create funding for healthcare subsidies and a stable healthcare subsidy system for patients with rare and incurable diseases (Japan Intractable Diseases Information Center). Research in the field of rare and incurable diseases has made great progress due to this wave of government support. The Japan Agency for Medical Research and Development invested in rare and incurable diseases’ research (Japan Agency for Medical Research and Development (AMED), 2017), and the Initiative on Rare and Undiagnosed Diseases was created as a platform for research and treatment of these conditions (Adachi et al., 2017; Adachi et al., 2018), thus generating a relatively large amount of new research in the field.

The fourth limitation is management of protocol papers. Because the approval requirements for core clinical research hospitals include protocol papers, a total of 44 protocol papers have been included in this study (44/580, 7.6%). Although these have several benefits, such as deterring publishing bias, preventing similar research, and giving hope to patients regarding the possibility of new or innovative treatments, protocol papers are published at the start of research, prior to result generation. Therefore, their quality should be evaluated separately from that of result-generating publications. We performed additional analyses with the data, excluding the 44 protocol papers. The results revealed a trend similar to that of the overall results. However, an analysis of protocol publications alone was not performed this time because of the limited number available, which was 44.

The fifth limitation is the evaluation of open access journals, which are known to have a higher citation impact than closed journals (Piwowar et al., 2018). Future studies should consider the handling of open access journals.

Finally, the relation between paper quantity and quality was only assessed for interventional clinical trials in this study. To address this issue, a subsequent study targeting both interventional and observational studies is currently being planned.

## Conclusions

This study revealed that the quantity of papers published by an institution does not necessarily correspond to their quality. When assessing an institution’s ability to execute clinical research, assessment of paper quality should be included alongside assessment of the number of published papers.

## Data Availability Statement

The datasets analyzed in this manuscript are not publicly available. Requests to access the datasets should be directed to Yuji Nishizaki, ynishiza@juntendo.ac.jp

## Author Contributions

RU and YN designed this study in whole and drafted this manuscript. RU contributed to data collection. NaoY and SN contributed to statistical analysis. YH, SS, TO, SY, KM, NaoY, MN, KF, SN, YS, NatY, and PD provided advice on the interpretation of results. YN and HD provided a critical revision of the manuscript for intellectual content and gave the final approval for the submitted manuscript. All authors have read and approved the final manuscript.

## Funding

The study was supported by a grant from the Japan Agency for Medical Research and Development (AMED) under Grant Number JP181k1903001 from April 1, 2018 to March 31, 2019.

## Conflict of Interest

The authors declare that the research was conducted in the absence of any commercial or financial relationships that could be construed as a potential conflict of interest.
